# Inuloxin A Inhibits Seedling Growth and Affects Redox System of *Lycopersicon esculentum* Mill. and *Lepidium sativum* L.

**DOI:** 10.3390/biom12020302

**Published:** 2022-02-12

**Authors:** Alessandra Villani, Maria Chiara Zonno, Silvana de Leonardis, Maurizio Vurro, Costantino Paciolla

**Affiliations:** 1 Department of Biology, University of Bari Aldo Moro, Via E. Orabona 4, 70125 Bari, Italy; alessandra.villani@uniba.it (A.V.); silvana.deleonardis@uniba.it (S.d.L.); 2 Institute of Sciences of Food Production, National Research Council, Via G. Amendola 122/0, 70126 Bari, Italy; mariachiara.zonno@ispa.cnr.it (M.C.Z.); maurizio.vurro@ispa.cnr.it (M.V.)

**Keywords:** ascorbate, garden cress, glutathione, Inuloxin A, redox systems, seed germination, thiol proteins, tomato

## Abstract

Allelochemicals are considered an environment-friendly and promising alternative for weed management, although much effort is still needed for understanding their mode of action and then promoting their use in plant allelopathy management practices. Here, we report that Inuloxin A (InA), an allelochemical isolated from *Dittrichia viscosa*, inhibited root elongation and growth of seedlings of *Lycopersicon esculentum* and *Lepidium sativum* at the highest concentrations tested. InA-induced antioxidant responses in the seedlings were investigated by analysing the contents of glutathione (GSH) and ascorbate (ASC), and their oxidized forms, dehydroascorbate (DHA), and glutathione disulphide (GSSG), as well as the redox state of thiol-containing proteins. An increase in ASC, DHA, and GSH levels at high concentrations of InA, after 3 and 6 days, were observed. Moreover, the ASC/DHA + ASC and GSH/GSSG + GSH ratios showed a shift towards the oxidized form. Our study provides the first insight into how the cell redox system responds and adapts to InA phytotoxicity, providing a framework for further molecular studies.

## 1. Introduction

The sessile lifestyle of plants has required the development of sophisticated and complex strategies for their adaptation to a wide range of environmental cues, including biotic and abiotic factors [1,2]. These strategies mainly consist of biosynthesis of secondary metabolites, which allow plants to compete for limited space, light, water, and nutrients, and increase production of enzymatic and non-enzymatic antioxidants primarily involved in counteracting the damaging effects of oxidative stress. Plant-derived secondary metabolites involved in plant-defence and -interactions, commonly referred to as allelochemicals, exert stimulatory or inhibitory effects on the growth and development of neighbouring organisms, including plants, insects, and pathogens [3,4,5,6]. The growing environmental and climate concern has increased interest in new efficient and eco-friendly approaches for weed and pest control, and for a sustainable agricultural management [7,8].

Allelochemicals may represent a valuable tool for developing environmentally friendly products, and several studies have been conducted that identify the potential herbicide, fungicide, and pesticide effects of secondary metabolites produced by several plants [4,7,9,10,11].

Over the last decades, many allelochemicals have been identified and for some of them the biological role has been also studied [12,13,14,15]. Allelochemicals belong to 14 classes according to their biosynthetic origin, including the more common flavonoids, simple phenolic acids, quinones, mono- and sesquiterpenes, and alkaloids [16,17].

Recently, numerous sesquiterpenes, specifically sesquiterpene lactones, were described and tested in standard target species as promising allelochemicals with herbicidal activity [5,18]. Among the investigated plants, *Dittrichia viscosa* (L.) Greuter (formerly named *Inula viscosa* (L.) Aiton) was widely described as a source of flavonoids, sesquiterpenes lactones, acids, and glycolipids showing phytotoxic and allelopathic effects [18,19,20,21]. This perennial weed belonging to the family Asteraceae and widespread throughout Europe, Africa, and Asia, has been used in traditional medicine for its anti-inflammatory, antifungal, antiulcerogenic, and antioxidant activity [19,22]. Although several studies have investigated the biological activities of extracts of this plant, few studies have focused on its herbicidal properties and its potential use in weed management practices [18,23].

Four bi- and tri-cyclic sesquiterpene lactones, named Inuloxins A–D extracted for the first time from the aerial parts of *Inula viscosa*, were described by spectroscopic and chemical methods and tested for their phytotoxic features, causing inhibition of seed germination of the parasitic plants *Orobanche crenata* Forsk. and *Cuscuta campestris* Yunck. [18]. The modest solubility of Inuloxin A (InA), which could have compromised its bioavailability and application in pest management, has been overcome by encapsulation with β-cyclodextrins with different methods [24]. The resulted complexes were characterized by different techniques and assayed for their bioactivity, showing the complete solubility in water and the ability to completely inhibit seed germination of *Phelipanche ramosa* (L.) Pomel., suggesting the use of InA as a novel natural herbicide for parasitic plant management. Although some studies have examined the potential role of InA as herbicide, causing necrosis of leaves, inhibition of germination of seeds and rootlet elongation [18,21,23,24,25], these studies have not explored the mechanism of action of this allelochemical. Understanding the molecular mode of action is a critical point for further exploring the practical use of allelochemicals as natural agrochemicals and to ascertain their possible no-target effects.

There is evidence in the literature for the involvement of ROS in the inhibition of root elongation, mediated by allelochemical or other abiotic stress [26,27,28]. A recent study, aimed at elucidating the response of *Panax notoginseng* (Burk) F.H. Chen to the triterpenoid saponins ginsenoside Rg1 via transcriptomic and cellular approaches, showed that some genes involved in plant growth and development were down-regulated after the roots were exposed to the allelochemical [29]. Furthermore, there is evidence that allelochemicals can cause oxidative stress in target plants and induce responses of the cellular antioxidant defence system [13,30,31]. Among the antioxidant metabolites, ascorbate (ASC) and glutathione (GSH) play a key role in defence system against biotic and abiotic stress [32,33]. These metabolites are both involved, together with some antioxidant enzymes, in the ASC-GSH cycle.

Therefore, in this study we explored the potentiality of the allelochemical InA as a natural herbicide for weed management, by assessing the effect of different concentrations of InA on the cell redox state of two target plant species, *Lycopersicon esculentum* and *Lepidium sativum*. In particular, non-enzymatic primary antioxidants in plants, such as ASC, GSH, and their oxidized forms, dehydroascorbate (DHA), and glutathione disulphide (GSSG), were analysed. The regeneration rates of ASC and GSH, expressed as ratios ASC/DHA + ASC and GSH/GSSG + GSH, respectively, were also obtained for evaluating the effect of InA on the efficiency of the ASC-GSH cycle. Moreover, the effect of InA on weight and root elongation, as well as the thiol-redox profiles in proteins were examined.

## 2. Materials and Methods

### 2.1. Plant Materials and Chemicals

Tomato (*Lycopersicon esculentum* Mill. var. *marmande*, Solanaceae) and garden cress (*Lepidium sativum* L., Brassicaceae) seeds were purchased from Sementi Gianfranco Fuscello (Andria, Italy) and Emanuele Larosa Seeds (Andria, Italy), respectively. These plant species were used as the model because of their well-known high germinability and good repeatability of root length measurements [24]. InA was extracted, purified, and provided by Professors Marco Masi and Antonio Evidente [18]. All reagents used in this study were of the highest grade available.

### 2.2. Effect of InA on Rootlets and Biomass

The effects of InA on the inhibition of the seed germination on the two selected species according to the procedure previously described by Vurro et al. [34] with some modification. Briefly, InA was dissolved in methanol and then diluted in methanol-water (2:98, *v*/*v*) to obtain the final concentrations of 10^−3^ M, 10^−4^ M and 10^−5^ M. Seeds (0.5 g) of *Lycopersicon esculentum* and *Lepidium sativum* were sanitized with 1% sodium hypochlorite solution (*v*/*v*) for 10 min, soaked with distilled water for 2 h, and placed into Petri dishes (12 cm diameter) on one layer of filter paper (Whatman Grade 4) moistened with 3 mL of distilled water to allow the germination. The Petri dishes were incubated at 25 °C in the darkness for 2 (*L. sativum*) and 4 (*L. esculentum*) days, respectively. Then, batches of ten seedlings (one batch for each treatment), chosen for homogeneity and health, were transferred to the filter paper in smaller Petri dishes (6 cm diameter). Each filter was moistened with 1 mL of InA solution at the above-described concentrations. Each treatment was replicated three times. Plates were placed in a growth chamber at 25 °C with a photoperiod of 12 h light/12 h dark for 3 and 6 days. Methanol (2% in water) was added to control seedlings. After 3 and 6 days, the rootlet length and weight were measured.

### 2.3. Determination of Ascorbate and Glutathione Pools

Treated and control seedlings (0.5 g) were homogenized with four volumes of cold 5% metaphosphoric acid in a porcelain mortar. The homogenate was centrifuged for 15 min at 20,000× *g* (4 °C) and the supernatant was collected and immediately assayed for ascorbate, dehydroascorbate, glutathione, and glutathione disulphide determination according to Zhang and Kirkham [35].

### 2.4. Determination of Total Proteins and Protein Thiol Labelling

Six day-old seedling of *L. esculentum* and *L. sativum* (0.5 g) were ground to a powder in liquid nitrogen with a mortar and pestle. SH-groups of proteins were labelled with monobromobimane (mBBr) according to Buchanan et al. [36] and Paciolla et al. [37], with some modification. One mL of 0.1 M Tris-HCl buffer (pH 7.9) containing mBBr 2 mM, (previously dissolved in acetonitrile), was added to each sample and homogenate for 1 min. Then, 25 µL of 100 mM 2-mercaptoethanol were added to 200 µL of mBBr-labelled sample for stopping the reaction and derivatizing the excess of mBBr. The protein assay was carried out as described in Mastropasqua et al. [38]. For the visualization of the thiol proteins, 100 µg of proteins of each sample were loaded on sodium dodecyl sulphate (SDS) gel (10% T, 4% C). SDS-PAGE was prepared according to Laemmli [39]. After electrophoresis run, gels were incubated with 12% (*w*/*v*) trichloroacetic acid solution for 1 h to fix the proteins and then with methanol:acetic acid:water (4:1:5 *v*/*v*) solution for 8–10 h to remove the mBBr excess. The fluorescence of mBBr-SH-groups was visualized under 365-nm UV light and photographed. The resulting fluorescence emission is indicative of the relative thiol/disulphide state of proteins analysed. The intensity of fluorescent bands was analysed with the UTHSCA Image Tool software.

### 2.5. Statistical Analyses

All data were statistically analysed using one-way analysis of variance (ANOVA) followed by Tukey’s post-hoc test, whenever significant differences were found (*p* ≤ 0.05). Statistical analyses and graphical representations were performed on GraphPad Prism software version 9.0.0 for Windows (GraphPad Software, San Diego, CA, USA)). Statistical significance was accepted at *p* < 0.01 (*), *p* < 0.001 (**), and *p* < 0.0001 (***). The data presented are the mean of at least 3 different replicates. The error bars represent standard deviation of the means.

## 3. Results and Discussion

Over the last decades, the increasing herbicide-resistant weeds and public concern about human health and the environment caused by the widespread use of synthetic herbicides, has made the use of sustainable weed management mandatory [40,41]. Much effort has been focused on natural compounds with an emphasis on new chemicals involved in plant–plant interactions for the discovery of potential herbicides [15,42]. Although a growing number of new allelochemicals have been described over the years, understanding the mechanism of action as herbicide and the ecotoxicological impact of those target molecules remain a major challenge [43,44].

In this study, we analysed the effect of various concentrations of InA on plant biomass and root growth, as well as the activity of some antioxidants involved in the detoxification and balance of cell redox state. InA concentrations used in the experimental design (see Section 2.2) were selected according to previous studies conducted on other plant species [18,24].

The root length and seedlings weight in *Lycopersicon esculentum* and *Lepidium sativum* exposed to three concentrations of InA, are presented in Table 1 and Table 2, respectively.

**Figure 1:**
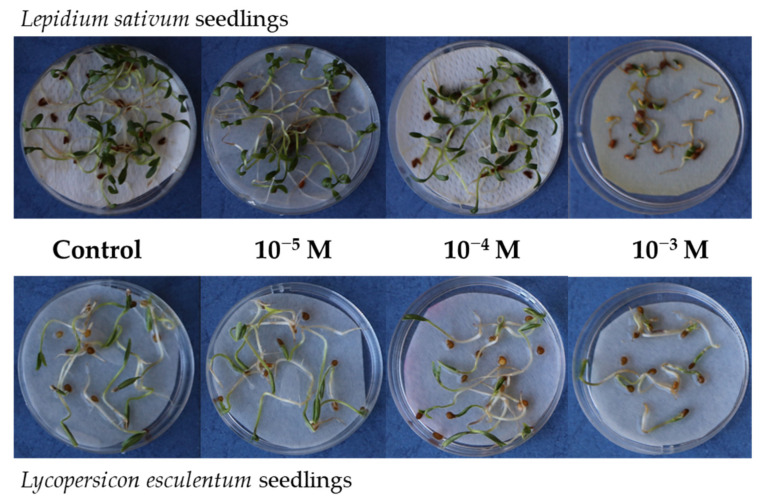
Morphological differences in seedlings of *Lepidium sativum* and *Lycopersicon esculentum* 6 days after the treatment with InA at three different concentrations.

Both plants showed significant inhibition of root elongation and biomass production when treated with the highest concentration of InA (10^−3^ M). After 6 days, at concentration of 10^−4^ and 10^−3^, *L. sativum* showed the highest inhibition of root growth (Table 1). Total plant biomass was significantly reduced after 3 and 6 days when exposed to the highest concentration of InA, while at 10^−4^ and 10^−5^ M the allelochemical had no or slight effect on the growth of tomato and garden cress seedlings (Table 2). The dose effect of InA has previously been observed in other plant species [18,24]. InA completely inhibited the germination of *Cuscuta campestris* (field dodder) and *Orobanche crenata* (broomrape) at concentrations of 10^−3^ M and 10^−4^ M, whereas inhibition activity was lost when the concentration of 10^−5^ M was tested [18]. However, at a lower concentration of InA (10^−6^ M), *Phelipanche* *ramosa* rootlets showed 99% reduction in length compared to the control [24]. Overall, our results are consistent with previous observations showing increased inhibition of root length with greater exposure concentrations of selected allelochemicals [5,28,30], although it must be highlighted that effect of InA on seed germination is species specific. Six days after the treatment with InA at 10^−3^ M, seedlings of both plant species showed significant morphological differences respect to the control (Table 1 and Table 2, Figure 1). The cotyledons were developed whereas the length of roots was inhibited and there was a marked decrease in survival with severe turgor loss (Figure 1). On the other hand, the seedlings treated with lower concentrations of InA, did not show severe symptoms, highlighting the ability of plants to counteract the phytotoxicity of the molecule. The survival of the seedlings could be due to metabolic rearrangements induced by intracellular ascorbate (ASC) and glutathione (GSH) increase, two primary antioxidant molecules in plant involved in regulation of cell redox balance.

Plants have adapted several strategies to ameliorate oxidative toxicity and sustain ROS homeostasis by modulating antioxidant chemicals, including enzymes and low molecular weight compounds [45]. Among these molecular compounds, ASC and GSH play a crucial role in redox homeostasis and signalling because of their high and widespread cell concentrations, interaction with specific enzymes that couple them to peroxide metabolism, and the relative stability of the corresponding oxidized forms [45,46]. For this reason, in this study we have investigated the effect of InA on the ASC and GSH pools. ASC is an essential component of plant antioxidant system, and it can spontaneously provide electrons to radical species alleviating the cellular oxidative stress caused by aerobic metabolism, biotic and abiotic stress [33]. Due to its redox potential (from +0.40 to +0.50 V) [47], ASC can quickly reduce the ROS, both indirectly contributing to the lipid radical-scavenging and then avoiding lipid peroxidation and preventing cell injury prior to activation of antioxidant enzymes, and directly by scavenging tocoferoxyl radicals, lipid peroxides, and oxidized metal ions [33,48]. Dehydroascorbate (DHA) was observed to accumulate in the apoplast after oxidative stress, and to inhibit the enzyme activity and root growth [37,49,50]. Additionally, DHA can be alternatively reduced by glutathione- ferredoxin- and NAD(P)H-dependent pathways demonstrating a metabolic link between the NAD(P)-dependent redox system and the low molecular weight antioxidants [51].

**Figure 2:**
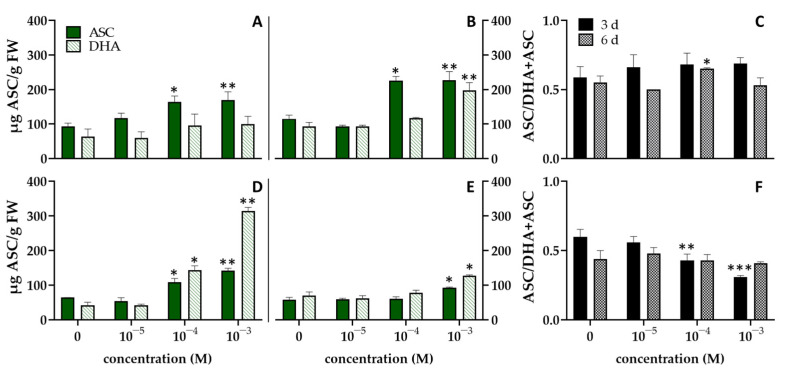
Effects of different concentration of InA on the contents of ascorbate (ASC) and dehydroascorbate (DHA) after 3 (**A**,**D**) and 6 days (**B**,**E**), and the ratio of ASC/DHA + ASC (**C**,**F**) of *Lepidium sativum* (**A**–**C**) and *Lycopersicon esculentum* (**D**–**F**) seedlings. Vertical bars indicate SD of three replicates in each treatment group. * Statistical significance at *p* < 0.01 (*), *p* < 0.001 (**), and *p* < 0.0001 (***). FW: fresh weight.

The effects of InA on the content of ASC in *L. esculentum* and *L. sativum* are shown in Figure 2.

Seedlings of *Lepidium sativum* showed levels of ASC higher than *Lycopersicon esculentum* both at 3 and at 6 days. As shown in Figure 2A,B, ASC content of seedlings of *L. sativum* treated with 10^−4^ and 10^−3^ M of InA was significantly higher (*p* ≤ 0.05) with respect to the control. DHA content did not change significantly except after 6 days at concentration of 10^−3^ M (Figure 2B). A significant increase in the ASC/ASC + DHA ratio occurred only at 10^−4^ M after 6 days (Figure 2C). The seedlings of *L. esculentum* treated with the higher concentration of InA, showed a significant (*p* ≤ 0.05) increase in ASC content at both times, whereas at lower concentration (10^−4^ M) the increase was significant (*p* ≤ 0.05) only at 3 days (1.68 times more than controls) (Figure 2D,E). A similar trend was observed in DHA levels. After 3 days, the ASC/ASC + DHA ratio decreased with increasing InA concentration, showing, at the highest concentration, a value 2 times lower than the control, whereas after 6 days of treatment, the ratio did not change significantly (Figure 2F). Overall, the increase in ASC in seedlings of *L. sativum*, at both exposure times, demonstrated that antioxidative defence responded to the tested concentrations of InA. However, seedlings of *L. esculentum*, showed higher levels of the oxidized form (DHA) than the reduced form (ASC) and the subsequent shift of the ratio towards the oxidized form (Figure 2D–F). As reported in previous studies higher concentration of the allelochemical could inhibit the ASC regeneration by down-regulating the activity of some ASC-GSH cycle enzymes [29,52,53].

**Figure 3:**
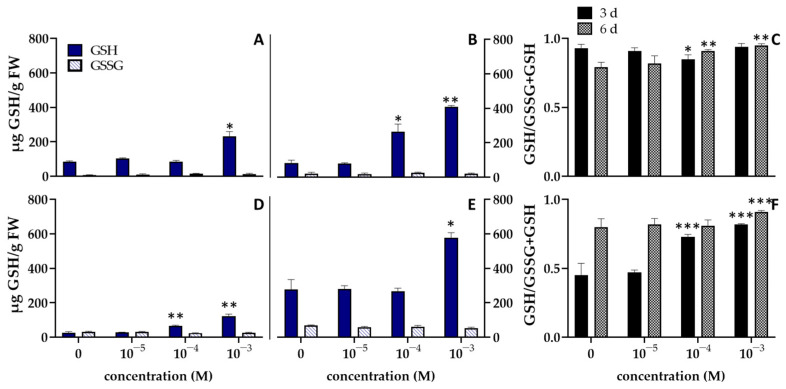
Effects of different concentration of InA on the contents of glutathione (GSH) and glutathione disulphide (GSSG) after 3 (**A**,**D**) and 6 days (**B**,**E**), and the ratio of GSH/GSSG + GSH (**C**,**F**) of *Lepidium sativum* (**A**–**C**) and *Lycopersicon esculentum* (**D**–**F**) seedlings. Vertical bars indicate SD of three replicates in each treatment group. * Statistical significance at *p* < 0.01 (*), *p* < 0.001 (**), and *p* < 0.0001 (***). FW: fresh weight.

In regard to glutathione (GSH), it is known that it is involved in storage and transport of reduced sulphur, gene expression regulation, pathogen resistance and, response to abiotic stress [46,54]. The contents of GSH, glutathione disulphide (GSSG), and the ratio of GSH/GSH + GSSG in the two plant species exposed to InA, are shown in Figure 3.

In both the tested species of seedlings, the highest concentration caused a significant (*p* ≤ 0.05) increase of GSH after 3 and 6 days. However, in *L. sativum* the GSH content was more than 2 and 4-fold higher after respectively 3 and 6 days than control, whereas in *L. esculentum* an opposite trend was observed, showing a more marked increase of GSH level at 6 days (Figure 3). Plants treated with 10^−4^ M of InA showed a significant increase of GSH level after 6 days (*L. sativum*, Figure 3B) and 3 days (*L. esculentum*, Figure 3D). In contrast, InA had little or no effect on GSSG level in all treatments analysed. Consequently, the GSH/GSH + GSSG ratio was significantly decreased in the *L. sativum* seedlings treated with 10^−4^ M of InA after 3 days, whereas after 6 days there was a significant increment of the ratio value from 0.79 in control to 0.91, and 0.95 in the seedlings treated with 10^−4^ and 10^−3^ M of InA, respectively (Figure 3C).

The GSH/GSH + GSSG ratio in *L. esculentum* seedlings significantly increased after 3 days, ranging from 0.45 in control to 0.73, and 0.82 in the samples treated with 10^−4^ and 10^−3^ M, respectively (Figure 3F). After 6 days, a significant increment was observed only with the highest concentration of InA.

**Figure 4:**
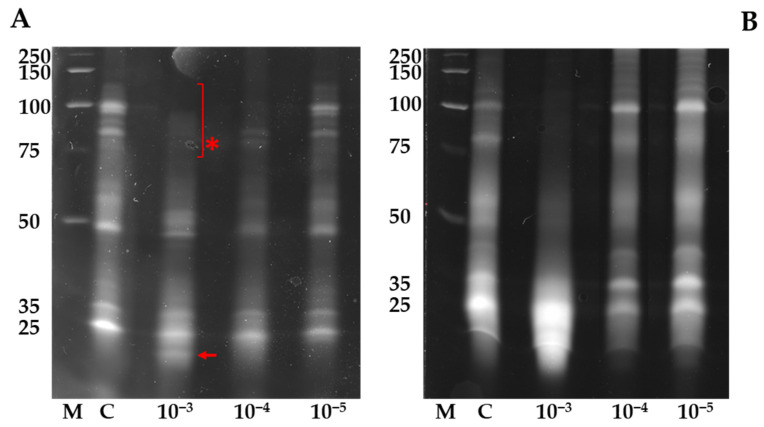
Analysis of the thiol/sulphide state of proteins labelled with mBBr. Seedlings of *Lepidium sativum* (**A**) and *Lycopersicon esculentum* (**B**) were analysed after 6 days of treatment with InA at different molar concentrations. M: molecular mass marker, whose sizes are indicated in kDa on the left; C: control. The presence of an additional fluorescent band (arrow) may be due to degradation of a larger thiol-containing protein that disappeared in the upper zone (asterisk).

The role of GSH is also crucial in radical scavenging and in preventing oxidative denaturation of proteins, by protecting their thiol group [54]. For this reason, we have also investigated the effect of the InA on the redox state of the proteins containing thiol groups. The thiol/disulphide status of proteins extracted from *L. sativum* and *L. esculentum* seedlings exposed to InA treatment for 6 days was detected by SDS-PAGE, using mBBr as fluorescent probe. The electrophoretic pattern of thiol-proteins group in seedlings of *L. sativum* and *L. esculentum* not- and treated is shown in Figure 4A,B, respectively.

Overall, the samples treated with InA showed lower fluorescence than the untreated sample (control), and fluorescence decreased with greater exposure concentrations (Figure 4A,B). In *L. sativum*, the average percentage decrease in fluorescence intensity, as compared to control, was −52%, −62% and −50%, in 10^−5^, 10^−4^ and 10^−3^ M InA treated samples, respectively (Figure 4A). This result indicated that InA treatment induced a shift towards the oxidized form of the protein thiol/disulphide redox state. However, at the highest concentration, an additional fluorescent band was observed (indicated by the arrow in Figure 4A), leading us to hypothesize that a new protein with thiol group could be synthesized by InA. It is well-known that stress conditions in plants can induce the disappearance or appearance of new proteins [55]. Furthermore, the presence of an additional fluorescent band can be due to degradation of a larger thiol-containing protein that disappeared in the upper zone of the protein thiol pattern (indicated by the asterisk in Figure 4A).

For *L. esculentum*, at the concentration of 10^−4^ M InA, the electrophoretic profile of protein thiols showed lower fluorescence compared to the control (−52%), whereas when 10^−3^ of InA was used, the presence of a fluorescent smear indicated a probable break down of the native thiol proteins (Figure 4B). It is noteworthy that the effect of InA on thiols was not evident when proteins were extracted after 3 days of treatment (data not shown) likely due to the high GSH levels through glutathiolation, a reversible post-translational modification consisting in a disulphide bond formation between a protein thiol and GSH.

## 4. Conclusions

In this work, we elucidated the response of seedlings of *Lycopersicon esculentum* and *Lepidium sativum* to InA, a secondary metabolite extracted from the foliar tissue of *Dittrichia viscosa* by analysing some molecular systems involved in the redox state balance. InA increased the content of non-enzymatic antioxidants ascorbate (ASC) and glutathione (GSH), involved in the ASC-GSH cycle. Metabolic perturbations were caused by changes in the pattern of the protein thiols that contributed to imbalance the cell redox state. The increased availability of ASC and GSH were important in defending against oxidative stress imposed by InA not only at early exposure, but also with extension of exposure time and increase of exposure dose. Although GSH and ASC are mainly involved in counteracting the cell redox imbalance, they play multifaceted roles with pleiotropic effects. Hence, further studies are needed to determine whether specific molecular mechanisms triggered by GSH and ASC, as well as by all the components included in the ASC-GSH cycle, could occur to preserve cellular redox balance. It remains to be investigated whether the protection of some key proteins from oxidation by glutathiolation are also operating as GSH-mediated protective effects. These results provide a framework for further molecular studies on the mechanism of action of InA and on the allelopathic abilities of *Dittrichia viscosa* to compete with other plants, limiting their development and germination.

## Figures and Tables

**Table 1:** Effect of different concentrations of InA on root length in seedlings of *Lycopersicon esculentum* and *Lepidium sativum*. Values are expressed as mean ± standard deviation (*n* = 3). * Statistical significance at *p* < 0.01 (*), *p* < 0.001 (**), and *p* < 0.0001 (***).

Days	Treatment	*Lycopersicon esculentum*		*Lepidium sativum*	
		Root Length (mm)	Root Length Inhibition (%) ^a^	Root Length (mm)	Root Length Inhibition (%) ^a^
3	10^−5^ M	34.8 ± 2.92	4.13	21.2 ± 0.19	9.97
	10^−4^ M	26.8 ± 1.82	26.2	19.9 ± 2.21	15.5
	10^−3^ M	14.1 ± 0.45 **	61.2	13.2 ± 1.07 ***	44.1
	control	36.3 ± 0.22	-	23.6 ± 1.56	-
6	10^−5^ M	65.1 ± 3.82	0.04	34.2 ± 1.63	0.91
	10^−4^ M	52.8 ± 5.94	18.9	20.3 ± 2.97 *	41.2
	10^−3^ M	13.9 ± 1.20 ***	78.6	3.73 ± 1.81 **	89.2
	control	65.2 ± 4.67	-	34.6 ± 2.76	-

^a^ Values are expressed as percentage of reduction respect to the control.

**Table 2:** Effect of different concentrations of InA on weight in seedlings of *Lycopersicon esculentum* and *Lepidium sativum*. Values are expressed as mean ± standard deviation (*n* = 3). * Statistical significance at *p* < 0.01 (*), *p* < 0.001 (**), and *p* < 0.0001 (***).

Days	Treatment	*Lycopersicon esculentum*		*Lepidium sativum*	
		Weight (mg)	Weight Inhibition (%) ^a^	Weight (mg)	Weight Inhibition (%) ^a^
3	10^−5^ M	18.8 ± 4.66	12.1	13.8 ± 0.44	14.4
	10^−4^ M	16.7 ± 0.55	22.3	11.6 ± 1.20 *	28.1
	10^−3^ M	13.3 ± 0.71 **	37.7	10.6 ± 0.28 **	34.4
	control	21.4 ± 0.84	-	16.1 ± 1.35	-
6	10^−5^ M	25.0 ± 3.30	0.62	17.6 ± 0.16	4.19
	10^−4^ M	22.7 ± 1.16	9.62	16.1 ± 0.41	12.3
	10^−3^ M	17.0 ± 0.35 **	32.4	9.16 ± 0.99 ***	50.2
	control	25.2 ± 0.74	-	18.4 ± 0.98	-

^a^ Values are expressed as percentage of reduction respect to the control.
